# Strategy to improve malaria surveillance system preventing transfusion-transmitted malaria in blood banks using molecular diagnostic

**DOI:** 10.1186/s12936-018-2486-z

**Published:** 2018-10-01

**Authors:** Sérgio Antônio Batista-dos-Santos, Daniel Roberto C. Freitas, Milene Raiol, Gleyce F. Cabral, Ana Cecília Feio, Marinete M. Póvoa, Maristela G. Cunha, Ândrea Ribeiro-dos-Santos

**Affiliations:** 1 Laboratório de Genética Humana e Médica, Universidade Federal do Pará, Belém, Pará CEP: 66075-110 Brazil; 2 Fundação Centro de Hemoterapia e Hematologia do Pará, Belém, Pará CEP: 660033-000 Brazil; 3 Universidade de Brasília, Brasília, Distrito Federal, Brasília, CEP: 70910-900 Brazil; 4 Instituto Evandro Chagas, Ananindeua, Pará CEP: 66087-082 Brazil; 5 Laboratório de Microbiologia e Imunologia, Universidade Federal do Pará, Belém, Pará CEP: 66075-110 Brazil

**Keywords:** Malaria, Molecular diagnostic, Blood donors, Transfusion-transmitted malaria, Hemovigilance, mt-qPCR

## Abstract

**Background:**

Malaria can be transmitted by blood transfusion through donations collected from asymptomatic or parasitic donors. The parasites are released into the bloodstream during its life cycle and will therefore be present in donated blood by infected individuals. All cases of transfusion-transmitted malaria (TTM) notified since 2005 in Brazil were fatal. A good screening tool for *Plasmodium* spp. detection in blood units must have a high detection threshold, and the prevention of TTM relies entirely on the exclusion of potentially infected donors. However, in Brazilian blood banks, the screening test relies on blood thick smears examination.

**Methods:**

The molecular diagnostic based on mitochondrial DNA (mtDNA) using real time PCR (mt-qPCR) was improved to detect *Plasmodium falciparum*, *Plasmodium vivax*, and standardized for use in *Plasmodium malariae.* The analytic sensitivity of this mt-qPCR methodology was performed using a sample of *P. vivax*.

**Results:**

The mt-qPCR was highly efficient, and the analytic sensitivity for *P. vivax* was determined (0.000006 parasites/µL). This method was tested to detect *P. vivax* and *P. falciparum* in individuals from two malaria-endemic areas in Brazil, Amazon region (Pará and Rondônia states), the samples were collected in 10 reference units of two blood banks (Pará/nine cities and Rondônia/Porto Velho), and parasites mtDNA were detected in 10 of 2224 potential blood donors (0.45%). In all 10 positive samples, only *P. vivax* was detected.

**Conclusion:**

Molecular diagnostic using mt-qPCR was effective in revealing infected potential donors with good perspectives to be applied as screening routine of asymptomatic carriers for preventing transfusion-transmitted malaria in blood banks.

## Background

Malaria is an infectious disease caused by parasites of the *Plasmodium* genus. In addition to being primarily transmitted by bites of female mosquitoes of *Anopheles* species, malaria is readily transmitted by blood transfusion through donations collected from asymptomatic or parasitic donors, especially those individuals with low parasite densities [1–4]. The parasite is released into the bloodstream during its life cycle and will therefore be present in blood donated by infected individuals [5–7].

The erythrocyte concentrate is the main blood component involved in transfusion-transmitted malaria (TTM), but there are reports of transmission by fresh frozen plasma, cryoprecipitate and platelets concentrate. When preserved in blood units, the parasite remains viable for a long period of time, even after storage. After transfusion, the disease begins in a matter of days or weeks, with severe and often fatal evolution, especially in immunodepressed individuals, young children, pregnant women, and people who have never been exposed to malaria parasites [6, 7].

Haemovigilance is required to identify and prevent the occurrence or recurrence of unwanted transfusion-related transmissions and to increase the safety, efficacy and efficiency of blood transfusions [8]. This vigilance system should include the monitoring of malaria in endemic areas, such as Africa, South America and some areas in Asia, to detect the five species of the *Plasmodium* genus that infect humans, *Plasmodium falciparum*, *Plasmodium vivax*, *Plasmodium malariae*, *Plasmodium ovale* and *Plasmodium knowlesi* [2, 9].

Malaria transmitted by blood transfusions has been described since the early 1900s. The first report on malaria as a consequence of an artery-to-vein blood transfusion was described in 1910 by Woolsey [5, 10]. However, the first case of TTM following the use of stored blood was described in 1941, and 1756 cases were reported in 49 countries between the years 1950 and 1972 [1]. In the last 40 years, the increased frequency of blood transfusion in medical practice and increase in travelling between countries in which malaria is absent and those where malaria is endemic reveals a clinical and public health problem [4, 8]. Moreover, the urbanization of malaria transmission areas in countries where the disease is endemic has contributed to an increased possibility of accidental transmission of *Plasmodium* infections through blood transfusion [11]. However, there is a paucity of officially registered data, which depends on appropriate tools for detection of malaria parasites.

An accurate diagnosis of *Plasmodium* infections is essential for malaria control or eradication [2, 9, 12]. Examination of thick blood smears is performed to detect positivity for *Plasmodium,* but this test has limited sensitivity to detect asymptomatic infections with low parasitaemia [13]. The lack of precise malaria diagnosis remains an important obstacle that limits the prompt detection and identification of *Plasmodium* species in the case of individuals with low parasitaemia [14].

In this context, for the first time, an innovative strategy was developed to detect the malaria parasites based on mitochondrial DNA (mtDNA) of malaria parasites, *P. falciparum* and *P. vivax* [15]. Efforts have been done to develop tools for disease control, especially to prevent TTM, by reporting the real possibility of transmission and the frequencies of contaminated samples from blood donors who resided in a large urban area within the malaria-endemic region [16]. After development and application of this novel method, other studies have reported the use of mtDNA for malaria diagnosis by nested PCR [17–21] or loop-mediated isothermal amplification (LAMP) [22, 23], thereby confirming the relevance of this innovative strategy of using mtDNA as a marker for malaria diagnostic tests [15].

Historically, the conventional malaria control has mostly taken aim at *P. falciparum*, and even its aggressive implementation has few lasting impacts upon endemic *P. vivax* [9, 12]. In part, it occurs because this resilient species requires multiple fronts of attack, which include the asymptomatic and sub-patent reservoirs [24, 25]. In general, transfusion malaria is not registered by national authorities. Since 2002, the official data reported only 4 cases transmitted by transfusions from the blood banks located in the Brazilian malaria-endemic area [11].

In Brazil, malaria is endemic in the Amazon region, where *P. vivax* is the main species. In the past two decades, the numbers of malaria cases caused by this parasite have varied between 100,000 and 500,000 per year, representing approximately 85% of all reported cases [26, 27]. Because *P. vivax* is able to develop latency stages (hypnozoites) in the liver, which leads to relapses, and its life cycle occurs inside the reticulocytes, these factors may contribute to an increase in the number of individuals with low parasite load, which are not usually detected by healthcare facilities [24, 25]. Moreover, in this endemic area, a high prevalence of asymptomatic *Plasmodium* infection has been reported [28]. This is an aspect that can lead to uncommon ways of transmission, such as TTM [1, 2].

In this study, the performance of molecular approach was improved for detection of malaria parasites via mitochondrial DNA as a careful screening test of blood samples from donors, and compared the more sensitive molecular technique with those used in haemovigilance as the best strategies for detecting *P. falcipar*um, *P. vivax* and *P. malariae*. In addition, for the *P. vivax* parasite, the prevalence of positive samples was determined as units for TTM occurrence upon the transfusion of blood or its components from infected donors living in malaria-endemic urban areas in Brazil.

## Methods

### Sample selection

Positive samples of *P. vivax* (22,000 parasites/µL) and *P. malariae* (400 parasites/µL) were obtained from patients with malaria. The positive sample of the 3D7 strain of *P. falciparum* was provided by the Evandro Chagas Institute/Ministry of health, Brazil. Samples of potential donors were collected in two blood bank centres. Among them, 1324 were obtained from nine reference units of the *Fundação Centro de Hemoterapia e Hematologia do Estado do Pará* (HEMOPA) and 900 were collected from blood donors in Porto Velho, the capital of the state of Rondônia. In samples collected in the state of Pará, the frequency of malaria parasites in potential donors was analysed according to age and annual parasite index (API), the gender ratio was approximately 4 males to 1 female.

### DNA extraction, quantification and mt-qPCR

DNA samples were extracted from 150 µL blood by phenol/chloroform followed by ethanol precipitation method, with modifications [29]. Then, DNA was quantified on NanoDrop™ ND 1000 (Uniscience), in 260 nm to 280 nm wavelength range and final concentration was adjusted to 100 ng/µL.

A qPCR was performed to determine the presence and absence of the malaria parasite mtDNA, based on primer extension and posterior hybridization with TaqMan probes on ABI Prism 7.500 sequence detection system (Life Technologies, CA, USA) version 2.0.6 software, according to the manufacturer’s instructions.

The probes were designed as described by Batista-dos-Santos et al. [16], targeting the Cytochrome C Oxidase gene (COX) in control samples of three species of *Plasmodium* evaluated in this study. The probes identified the species *P. falciparum* (COX III—access n. GI8346992 and M76611), *P. vivax* (COX I—access n. GI63022502). The qPCR assay amplified mtDNA which was a part of the sequence generated by the primers as published by Cunha et al. [15], and *P. malariae* (COX I—access n. KC175322.1), 3 different qPCR assays were performed.

As negative control, ultra-pure water previously tested for mitochondrial qPCR (mt-qPCR) was employed and a molecule of template DNA was used as endogenous positive control.

### Evaluation of the detection threshold of mt-qPCR for *Plasmodium vivax*

Evaluation of analytic sensitivity of the mt-qPCR methodology was performed using a sample of *P. vivax* previously evaluated and quantified. For this, a serial dilution was performed in ultra-pure water (DF 10) until the concentration of 1:100,000, in triplicate, as previously described by Gama et al. [30], using serial dilution for genomic DNA (dDNA). It was adapted for mitochondrial DNA, hence the results are sub-represented considering that the copy number of the mitochondrial genome is estimated in an interval of 20–150 copies [31, 32].

The aliquot of 150 µL of blood was withdrawn from the sample containing 200 parasites/mm^3^. After extraction, the pellet was precipitated in a volume of 50 µL (~ 600 parasites/µL). In addition, the solution was diluted to 1:1000 in ultra-pure water, which corresponded to approximately ~ 0.6 parasites/µL.

## Results

### Comparison of the mt-qPCR reactions to detect *Plasmodium malariae*, *Plasmodium falciparum* and *Plasmodium vivax*

**Fig. 1:**
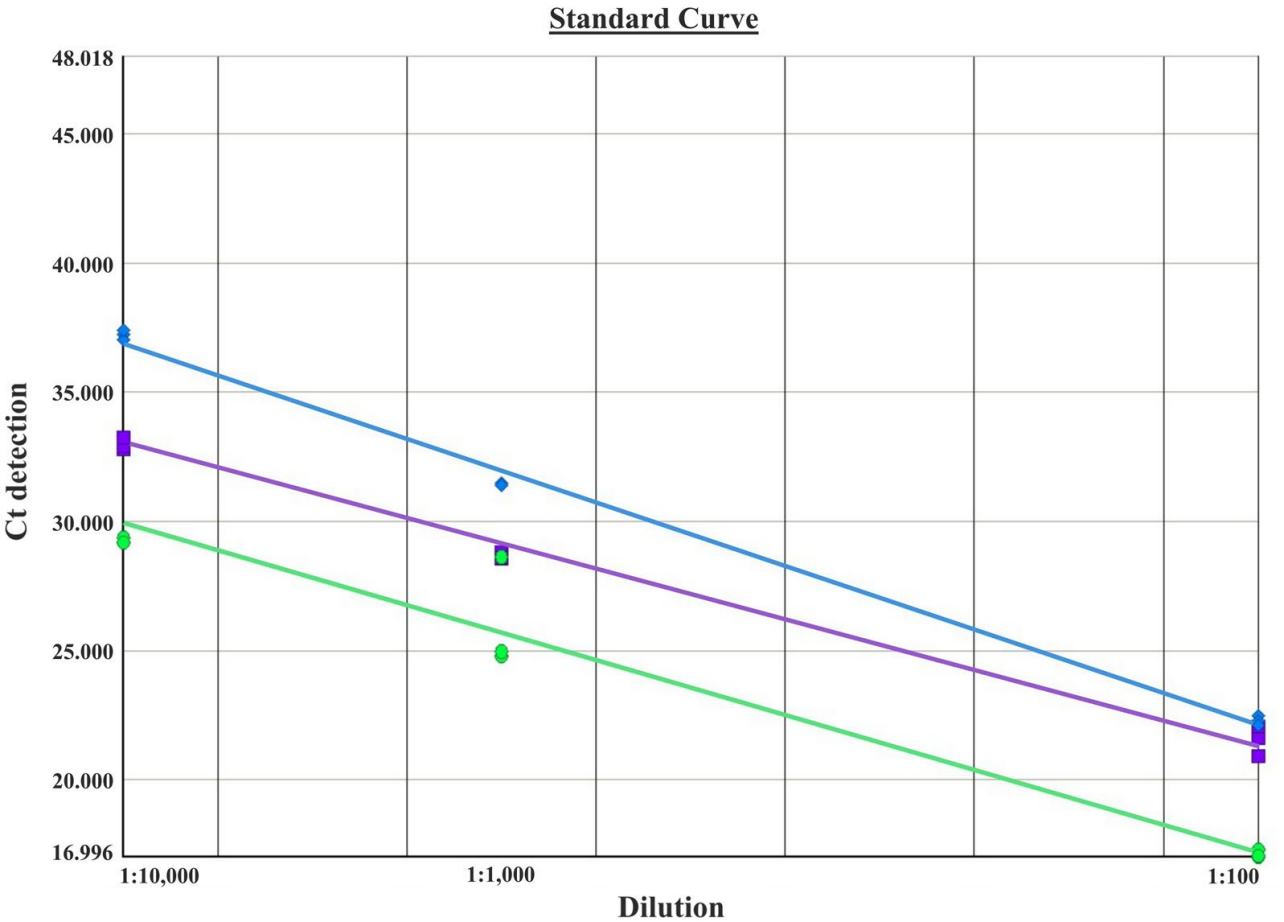
Comparison of the mt-qPCR reactions to detect *Plasmodium malariae, Plasmodium falciparum* and *Plasmodium vivax.* The amount of amplicons obtained from amplification of mtDNA from malaria parasites showed variability according to the parasitaemia density of each *Plasmodium* species tested. The DNA dilutions tested were 1:100, 1:1000 and 1:10,000. *P. malariae* (blue), *P. vivax* (purple) and *P. falciparum* (green)

Initially, mt-qPCR was standardized to detect *P. malariae,* and the detection threshold was determined in comparison with *P. falciparum* and *P. vivax* using this molecular diagnosis. The amount of amplicon obtained by the amplification of mtDNA from malaria parasites showed variability according to the parasitaemia density of each *Plasmodium* species tested. This assay showed good reproducibility when tested for all three dilutions of each sample (Fig. 1).

### Determination of the detection threshold of the mt-qPCR for molecular diagnosis of *Plasmodium vivax*

**Fig. 2:**
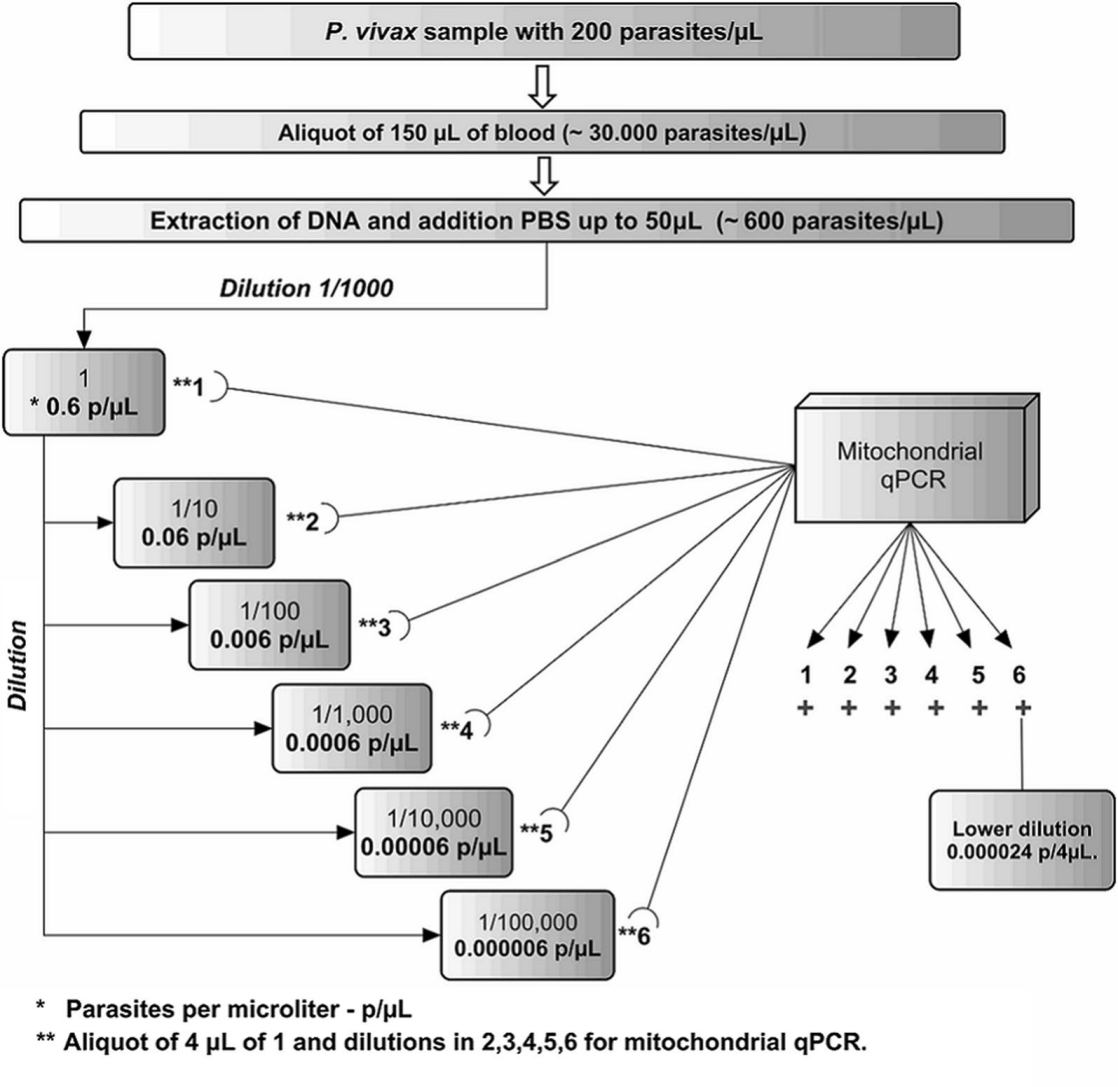
Determination of the *Plasmodium vivax* detection threshold by mt-qPCR. Serial dilutions were performed using 1.0 μL of the DNA solution (~ 0.6 parasites/μL) for obtained serial dilutions in ratio 10, with dilutions equivalent from 0.6 to 0.000006 parasites/μL. The same volume of each dilution was tested in triplicate to estimate the analytical sensitivity

For *P. vivax* sample, the detection threshold was estimated by analysing the blood collected from an infected individual. The parasitaemia was determined by the thick blood smear technique (200 parasites/µL). The serial dilutions were established as follows: (i) the first solution contained the equivalent of 0.6 parasites/μL; (ii) the second solution contained 0.06 parasites/μL; (iii) the third solution had 0.006 parasites/μL; (iv) the fourth solution had 0.0006 parasites/μL; (v) the fifth solution had 0.00006 parasites/μL; and, (vi) the sixth solution contained 0.000006 parasites/μL (Fig. 2).

The technique of amplification by mitochondrial qPCR was able to detect *P. vivax* mtDNA at all dilutions, even in the most diluted sample corresponding to 0.000006 parasites/µL (with CT of 38). The detection thresholds for the *P. falciparum* and *P. malariae* species were not determined.

### Percentages of positive samples from blood donors identified by qPCR detection of *Plasmodium falciparum* and *Plasmodium vivax* mtDNA

A total of 2224 samples collected from blood donors were tested by molecular diagnosis using mt-qPCR. All participants were permanent residents of two different states in the Brazilian Amazon region. Of these, 1324 were collected from different blood banks of the state of Pará, and 900 were from blood donors from Porto Velho, the capital of the state of Rondônia. Malaria parasites were detected in 10 of 2224 blood donors (0.45%). In all 10 positive samples, only *P. vivax* mtDNA was detected. The distribution of blood donors by city and the prevalence of detected malaria parasites is shown in Table 1. The positivity for *Plasmodium* was observed in only 3 blood banks, and the numbers of positive samples were 5, 3 and 2 for those collected in Abaetetuba (Pará), Santarém (Pará) and Porto Velho (Rondônia), respectively. Regarding samples collected in Porto Velho, *P. vivax* was detected using both molecular diagnostic methods (mtDNA and 18S rRNA). The percentage of positive samples was 3.0, 1.5 and 0.2% for the Abaetetuba, Santarém and Porto Velho donor groups, respectively. All tested samples were negative for *P. falciparum.*

**Table 1:** Baseline characteristics of the samples collected in blood banks of the Brazilian Amazon region and exposure to *P. vivax* and *P. falciparum* detected by molecular diagnosis

Blood banks located in Pará and Rondônia state	N	Age in years, median (range)	Males, %	API^a^	mt-qPCR positive N (%)	
					*P. falciparum*	*P. vivax*
Belém–Pará^b^	120	32 (18–59)	85.0	0.7 (low)	0 (0)	0 (0)
Castanhal–Pará	161	31 (19–59)	73.3	0.4 (low)	0 (0)	0 (0)
Marabá–Pará	193	30 (18–65)	75.6	5.9 (low)	0 (0)	0 (0)
Santarém–Pará	197	34 (20–60)	66.0	3.1 (low)	0 (0)	3 (1.5)
Abaetetuba–Pará	167	30 (19–62)	76.0	0.9 (low)	0 (0)	5 (3.0)
Redenção–Pará	129	34 (20–58)	71.3	0.15 (low)	0 (0)	0 (0)
Capanema–Pará	155	28 (19–60)	66.4	0.5 (low)	0 (0)	0 (0)
Tucuruí–Pará	33	32 (18–65)	60.6	35.6 (medium)	0 (0)	0 (0)
Altamira–Pará	169	26 (18–59)	75.7	22.7 (medium)	0 (0)	0 (0)
Porto Velho–Rondônia^c^	900	NA	NA	54.3 (high)	0 (0)	2 (0.2)
All samples	2224	30 (18–65)	73.1	–	0 (0)	10 (0.45)

*NA* not available

^a^Annual Parasite Index at the year of the survey with the respective classifications (low, medium or high) as reported by the Brazilian Ministry of Health (http://www.saude.gov.br/sivep_malaria)

^*b*^All donors from Pará state were selected to donation

^*c*^Among all samples collected in Rondônia state, only two were positive for *P. vivax* by molecular screening, 700 were approved as donors and 200 were excluded, all of them were tested in both molecular methods, the nested polymerase chain reaction (nested-PCR) for amplification of the 18S RNA and the real time PCR (qPCR) to amplify the parasites mtDNA

## Discussion

TTM is recognized in many countries where malaria transmission has been eliminated [6]. Although the frequency of this route of transmission is very low in non-endemic malaria areas, it is a substantial problem that involves the risk of receiving a unit of erythrocyte concentrate contaminated with parasites [7, 33]. Transfusion-associated cases of malaria have been reported in many parts of the world, including the USA [1, 3, 34], Canada [35], Spain [36], England [37], and France [38], Therefore, it should be noted that TTM may be implicated in the reintroduction of malaria into areas where it had previously been eradicated.

Conversely, the risk of transmitting malaria by blood transfusion in countries where parasite transmission is endemic can be high, and the healthcare systems clearly need alternative measures to prevented TTM, including tools for detecting the parasite or its components in the blood of potentially infected donors [2, 7, 11].

Although the exact numbers are unknown, the frequency of TTM has been estimated as 1 case per 4 million units donated in non-endemic areas [36, 37]. Moreover, it is worth considering that in the malaria-endemic areas, it is a health problem as reported for Africa [7, 39, 40], Asia [41] and South America, including Brazil, where infected donors were detected in 2 studies that used different molecular approaches to test samples collected in blood banks located in the Amazon region [16, 42]. Besides, TTM is a disease with a high lethality rate: all cases notified in Brazil since 2005 were fatal [43].

Malaria screening is mandatory in endemic regions and the directives of the Brazilian National Health Surveillance Agency (ANVISA) and the Brazilian Ministry of Health require high sensitivity tests. In spite of this, malaria screening in Brazilian blood banks relies on questionnaire data followed by microscopic examination of thick smears, which has several limitations, such as low counts of parasitaemia in asymptomatic patients which may lead to liability to human error [11].

In this study there was an improvement in the methodology developed by Cunha et al., which originally used a strategy based on mtDNA to amplify specific sequences of the Cytochrome C Oxidase genes, such as COXIII and COXI for *P. falciparum* and *P. vivax*, respectively. The conventional PCR methodology was changed to a more sensitive and semi-automated technique using one-step qPCR and the same mtDNA sequences. Additionally, mt-qPCR specific to *P. malariae* was standardized. The conventional PCR identified small amplicons of 273 pb (*P. falciparum)* and 290 pb (*P. vivax*) [15] allowing the parasite mtDNA amplification in a shorter time than in the classic nested-PCR based on ribosomal RNA, which has been used for molecular diagnosis of malaria over the past two decades [44].

This new methodology was applied for the detection of 3 *Plasmodium* species (*P. falciparum*, *P. vivax,* and *P. malariae*) more frequently found as the infecting agents among the 5 species that cause human malaria. The results confirmed that potentially infected volunteers can be among the donors. Although the frequency was low, it represents an even more complicated issue considering that each donation can be distributed over erythrocyte concentrates, platelet concentrates, or serum or plasma/platelets concentrates. *Plasmodium falciparum* and *Plasmodium vivax* tests were included, but only samples infected with *P. vivax* were detected. This can be explained by the fact that this is an area of higher incidence of *P. vivax*. It is well-characterized as an endemic area where *P. vivax* causes approximately 85% of malaria episodes registered by the Brazilian Health System [26, 27].

Another contribution was the qPCR designed specifically for detecting mtDNA of *P. malariae*. This innovative approach, although only preliminarily tested one positive sample, proved to be a suitable screening tool for identification of infected patients. The detection threshold curve varied depending on the infecting species. This suggests that *P. malariae,* which frequently occur in low parasitaemia, can also be detected by mt-qPCR. Future studies may be conducted to validate this assay in a cross-sectional study. In Brazil, it is necessary because there are certain issues with the transmission of *P. malariae* that need to be addressed, which include a high prevalence of *P. malariae* infections in the Brazilian Amazon endemic area [45], as well as a case of TTM after a transfusion of blood donated by an asymptomatic donor infected with this parasite [46]. In both studies, nested-PCR detecting rRNA was used for diagnosis.

The major advantage of using mitochondrial DNA (mtDNA) in malaria diagnosis as originally reported [15] is the largest copy number/cell of the parasite mitochondrial genome, from 20 to 150 copies/parasite [18, 31, 32]. The potentially infectious parasitaemia is largely variable in TTM cases. An estimated value is 10 parasites/unit in 250 mL of erythrocyte concentrate. In this case, a molecular diagnostic method to provide a trustworthy analysis should have a very low parasite detection threshold [33].

Another important point to be considered is the volume of blood needed to obtain the DNA, since the amount of processed specimen determines the limit of detection [33]. Some questions have been raised regarding the volume of blood that should be tested because only a small part of the donor’s total blood volume, which will be transferred to the recipient individual, is included in the molecular analysis. Hence, different concentrations of DNA obtained from *P. vivax*-infected patients were tested, and the estimated detection threshold was very low (0.000006 parasites/µL) when compared to the detection threshold of the nested PCR (0.004 parasites/µL) [36].

Two research groups discussed this issue and proposed a hypothetical detection threshold, using calculations that offered the hypothetical value as the threshold [33, 36]. Thus, a threshold could be established corresponding to the probability that analysis of a very small volume of the whole blood bag produces a reliable result that may serve as the basis for a conclusion regarding whether the screening could be positive in very low parasitaemia. The qPCR using mtDNA as template for identification of *P. vivax* proved that it was possible to reach the hypothetical detection threshold to detect parasitaemias as low as 2.2 × 10^−6^ parasites/µL, as described by Hänscheid et al. [33] in a commentary on the use of PCR for screening blood donors tested by Benito and Rubio [36]. Our tests showed that mt-qPCR detected *P. vivax* at 2.4 × 10^−5^ parasites/4 µL (6.0 × 10^−6^ parasites/µL) density by analysing DNA from a suitable blood volume, thus achieving analytical sensitivity very close to the value estimated almost two decades ago [33], that is, before the routine use of more sensitive molecular techniques such as real-time PCR.

Malaria induced by blood transfusions from asymptomatic carriers is a relevant problem of haemovigilance. A review of the duration of *P. falciparum* infections has illustrated how these infections have been studied for decades, and based on the data from these studies, it is possible to suggest that for blood transfusion-transmitted malaria, the inoculum should contain approximately 1000 parasites in a blood bag or bottle of approximately 500 mL. Thus, the transmission density approached an average of 2 parasites/mL [5]. A appropriate screening tool for malaria detection in blood units must have a high sensitivity [33]. It must allow the reduction of TTM risk as well as that of falsely deferred blood donors.

Finally, this analysis proved that it was possible to considerably improve the assay to detect the parasite mtDNA, compared to conventional PCR, where the detection threshold was 0.01 parasites/µL [15]. Using mt-qPCR, the achieved threshold was substantially lower, 0.000006 parasites/µL. This confirms the high efficiency of this methodology to detect parasite mtDNA (*P. vivax*) analysing only 150 µL out of a total of approximately 450 mL of blood.

Prevention of TTM relies entirely on the exclusion of potentially infected donors. However, it can be very difficult since in many cases, the donor screening process is not efficient and fails to obtain accurate information on previous exposure to malaria and identification of the infected donors. It represents a situation that can particularly occur for donors in blood banks of endemic-malaria areas [7, 11, 39].

Analysing the frequency of positive donor detection by molecular diagnostic tools for samples from blood banks from the malaria-endemic area in Brazil, it was identified only two studies up until the present that determined the prevalence of positive samples of *Plasmodium* species among donors that live in the Amazon region, which varies from 1 to 3%. Both studies included samples collected from donors of the blood banks of the Belém city, and the prevalence observed was similar, 1.34% [16] and 1% [42]. It should also be noted that in these studies with blood bank samples, it was only detected positives for a single species, *P. vivax* [16], while others reported mixed infection [42]. It can be suggested that mt-qPCR provides a more specific amplification. In a malaria-free area, a non-endemic state located outside the Amazon region, cases of positive samples from blood donors were registered [47]. This motivates an intense discussion that involves technical and epidemiological aspects and exposes how important it is to provide more accurate data, as well as to provide new highly sensitive tools for better explanations of this situation [47–49].

A suitable screening tool for malaria detection in blood units must have high sensitivity and specificity; it must be fast and reduce the risk of TTM [2, 4]. Considering the limitations of the thick blood smear as a screening method in blood banks, it is suggested that the cost-effectiveness of introducing a molecular diagnostic tool for *Plasmodium* species in the blood banks of endemic areas or in micro-regions should be evaluated in addition to the possibility of reducing the risks of TTM. Malaria diagnostic research provides a basis for applied advances in malaria surveillance [9, 13–15]. For this, the laboratorial blood screening can be a critical point in malaria eradication. In the case of TTM, one aspect that must be considered is the low parasitaemia of infected donors.

## Conclusions

In conclusion, qPCR is efficient for malaria molecular diagnostics, presenting promising results with good analytic sensitivity. It is a fast and easy molecular methodology to detect mtDNA of the three most frequent malaria parasites, presenting a potential for large-scale use in the prevention of transfusion-transmitted malaria by screening for potential donors as part of malaria haemovigilance in blood banks.
